# The coding complete genomic sequence of a feline panleukopenia virus detected from a domestic cat (*Felis catus*) in Chattogram, Bangladesh

**DOI:** 10.1128/mra.00209-24

**Published:** 2024-07-09

**Authors:** Jesmin Akter, Chandan Nath, Md Saddam Hossain, Md Ahaduzzaman

**Affiliations:** 1 Department of Medicine and Surgery, Chattogram Veterinary and Animal Sciences University (CVASU), Chattogram, Bangladesh; Katholieke Universiteit Leuven, Leuven, Belgium

**Keywords:** feline panleukopenia virus, epidemiology, genome sequence, phylogenetic analysis

## Abstract

The coding complete genome sequence of the feline panleukopenia virus (FPLV), detected from an indigenous cat in Bangladesh, has been determined. The genome spans 4,842 bp and contains four protein-coding genes. The genome will contribute to a comprehensive understanding of the genetic traits and evolutionary trends of FPLV.

## ANNOUNCEMENT

Feline panleukopenia virus (FPLV) is a non-enveloped, single-stranded DNA virus belongs to *Protoparvovirus carnivoran1* species of the genus *Protoparvovirus* in the family *Parvoviridae* (subfamily *Parvovirinae*). It is responsible for causing a highly contagious, life-threatening disease of domestic cats and wild felines (1, 2), known as feline panleukopenia or feline distemper. The disease is widely prevalent in many countries around the world and poses as an emerging and re-emerging threat (3). FPLV has an affinity for rapidly growing cells in the intestine, lymph nodes, and bone marrow, leading to symptoms such as fever, hemorrhagic gastroenteritis, and immunosuppression (2). The virus is closely related to mink enteritis virus and canine parvovirus (2).

A rectal swab sample was collected from a clinically ill indigenous domestic cat that tested positive for FPLV using a rapid antigen test kit (FPV Ag test cassette, Testsealabs, China) during a recent outbreak (October 2023) at the teaching veterinary hospital (TVH) of Chattogram Veterinary and Animal Sciences University (CVASU), Chattogram, Bangladesh (22°21′43.3″ N 91°48′15.8″ E). The sample was collected by the veterinarian for diagnostic purposes with the consent of the animal’s owner, following the sampling protocols and animal handling standards of TVH, CVASU. The sample was immediately transported to the lab in viral transport media (consisting of brain heart infusion broth, fluconazole, benzyl-penicillin, and gentamycin) and stored at −20°C. DNA extraction was performed using the Monarch genomic DNA purification kit (New England BioLabs, Inc., USA). Initially, the sample was tested using a conventional polymerase chain reaction (PCR) targeting the VP2 gene of FPLV (4) before being sent for complete genome sequencing to a biotechnology company (Macrogen, South Korea). The PCR amplicon library was prepared using the Nextera XT DNA library preparation kit with adapter sequences and primers (document #1000000002694v18, Illumina), and electrophoresis was conducted using the Agilent D5000 screen tape system. Sequencing was carried out using the Illumina system (Illumina HiSeq 4000) with paired-end, 2 × 150 bp sequencing.

The conventional PCR confirmed the sample as FPLV-positive. The WGS library QC results showed a concentration of genomic DNA at 7.27 ng/µL (11.57 nM). Following sequencing, a total of 29,378,162 reads were generated. The comprehensive genome assembly was conducted using the Unicycler v0.4.8 assembly process (5), and the polishing of the assembled genome was done using Pilon v1.23 (6), utilizing the paired read files with default parameters applied throughout (7). The annotation of the coding region of the genome was done using the ExPASy translate tool (8).

**Fig 1:**
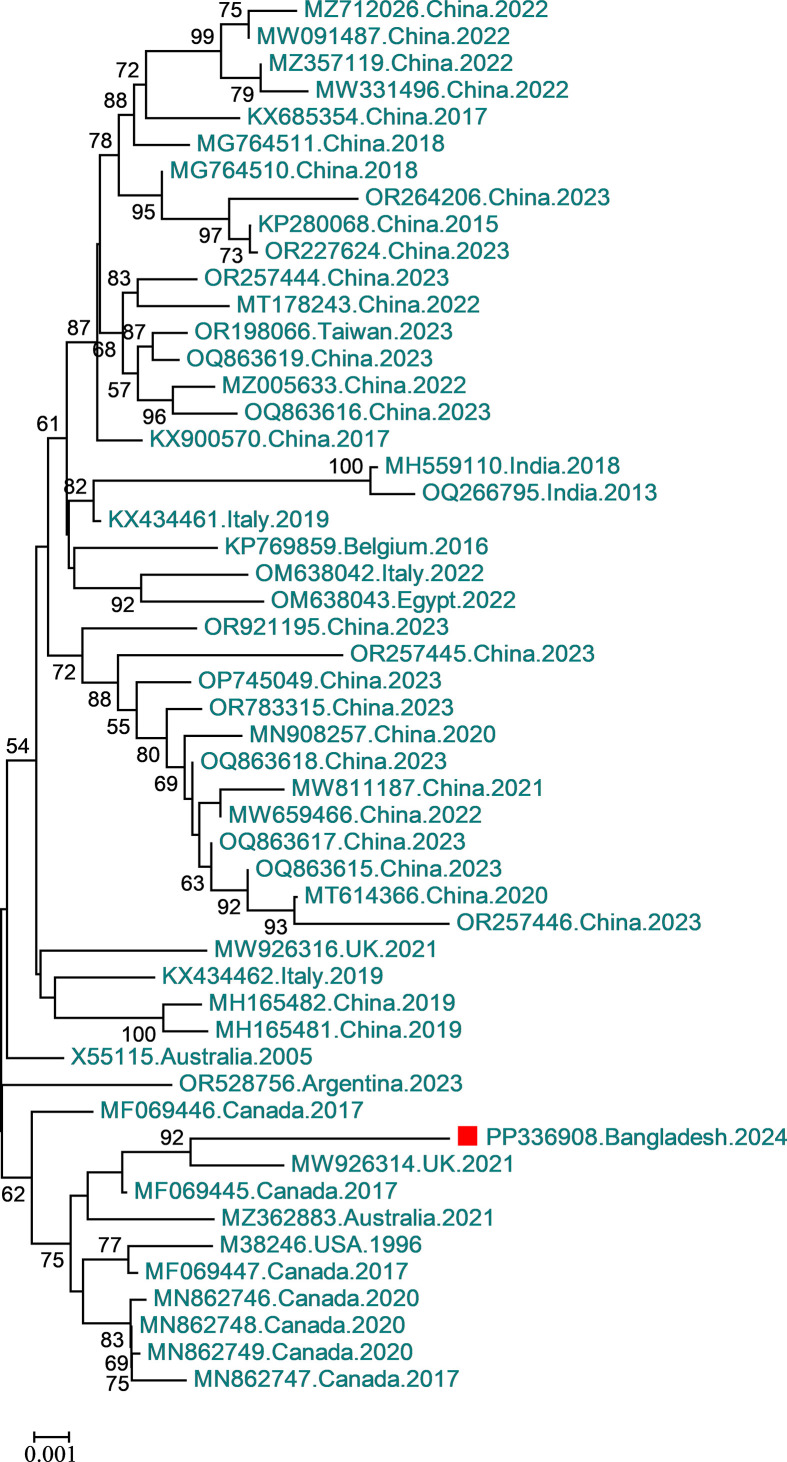
Phylogenetic analysis of FPLV is based on the nucleotide sequence of the coding complete genome. The FPLV genome in this study was compared to sequences in the NCBI GenBank nucleotide database using the BLASTn program. Similar sequences were retrieved, imported into MEGA v7, and aligned using ClustalW. A tree was generated using the neighbor-joining method in MEGA-7, employing the Tamura 3-parameter model with gamma distribution (9). The percentage of reliability for each node was determined via bootstrap analysis with 1,000 replicates. Bootstrap values below 50 are not shown. Data are presented as NCBI accession number, country, and year, with the sequence from this study marked as a red square.

The detected FPLV was designated CVASU/BD/50. The genome sequence was 4,842 bp long and had a GC content of 35.48%. Similar to other FPLVs, the FPLV detected in this study possesses two open reading frames (ORFs). ORF1 spans from 292 to 2,298 bp (2,007 bp, 668 aa), and the ORF2 spans from 2,392 and 4,560 bp (2,169 bp, 722 aa). There are two coding regions for non-structural (NS) proteins: NS1 (292–2,298 bp) and NS2 [join (292–551 bp, 2,024–2,261 bp)], and two coding regions for structural or viral capsid proteins (VP): VP1 [727 aa, CDs join (2,305–2,335 bp, 2,408–4,560 bp)] and VP2 (2,806–4,560 bp). The NS2 and VP2 sequences are completely contained within the NS1 and VP1 sequences, respectively, due to alternatively splicing. Phylogenetic analysis suggests that the FPLV in this study is closely clustered with the United Kingdom Cat 1 FPLV, but is slightly more divergent, with a percent identity of 98.92% (Fig. 1).

## Data Availability

The complete coding sequence of the FPLV has been deposited in GenBank under accession number PP336908 with the BioProject number PRJNA1081166 and SRA accession number SRR28110949.

## References

[1] Roozitalab A , Elsakhawy OK , Abouelkhair MA . 2023. Complete coding sequence of two feline panleukopenia virus strains isolated from domestic cats (Felis Catus)in tennessee, USA. Microbiol Resour Announc 12:e0043123. doi:10.1128/MRA.00431-23 37768073 PMC10586171

[2] Truyen U , Parrish CR . 1992. Canine and feline host ranges of canine parvovirus and feline panleukopenia virus: distinct host cell tropisms of each virus in vitro and in vivo. J Virol 66:5399–5408. doi:10.1128/JVI.66.9.5399-5408.1992 1323703 PMC289096

[3] Barrs VR . 2019. Feline panleukopenia: a re-emergent disease. Veterinary Clinics: Small Animal Practice 49:651–670. doi:10.1016/j.cvsm.2019.02.006 30967253

[4] Mochizuki M , Horiuchi M , Hiragi H , San Gabriel MC , Yasuda N , Uno T . 1996. Isolation of canine parvovirus from a cat manifesting clinical signs of feline panleukopenia. J Clin Microbiol 34:2101–2105. doi:10.1128/jcm.34.9.2101-2105.1996 8862565 PMC229197

[5] Wick RR , Judd LM , Gorrie CL , Holt KE . 2017. Unicycler: resolving bacterial genome assemblies from short and long sequencing reads. PLoS Comput Biol 13:e1005595. doi:10.1371/journal.pcbi.1005595 28594827 PMC5481147

[6] Walker BJ , Abeel T , Shea T , Priest M , Abouelliel A , Sakthikumar S , Cuomo CA , Zeng Q , Wortman J , Young SK , Earl AM . 2014. Pilon: an integrated tool for comprehensive microbial variant detection and genome assembly improvement. PLoS One 9:e112963. doi:10.1371/journal.pone.0112963 25409509 PMC4237348

[7] Snyder EE , Kampanya N , Lu J , Nordberg EK , Karur HR , Shukla M , Soneja J , Tian Y , Xue T , Yoo H , et al. . 2007. PATRIC: the VBI pathosystems resource integration center. Nucleic Acids Res 35:D401–6. doi:10.1093/nar/gkl858 17142235 PMC1669763

[8] Artimo P , Jonnalagedda M , Arnold K , Baratin D , Csardi G , de Castro E , Duvaud S , Flegel V , Fortier A , Gasteiger E , Grosdidier A , Hernandez C , Ioannidis V , Kuznetsov D , Liechti R , Moretti S , Mostaguir K , Redaschi N , Rossier G , Xenarios I , Stockinger H . 2012. ExPASy: SIB bioinformatics resource portal. Nucleic Acids Res 40:W597–603. doi:10.1093/nar/gks400 22661580 PMC3394269

[9] Kumar S , Stecher G , Tamura K . 2016. MEGA7: molecular evolutionary genetics analysis version 7.0 for bigger datasets. Mol Biol Evol 33:1870–1874. doi:10.1093/molbev/msw054 27004904 PMC8210823

